# Ailanthone Inhibits Proliferation, Migration and Invasion of Osteosarcoma Cells by Downregulating the Serine Biosynthetic Pathway

**DOI:** 10.3389/fonc.2022.842406

**Published:** 2022-02-03

**Authors:** Yawen Zhang, Runze Gong, Yong Liu, Xipeng Sun, Jinrong Liang, Yan Zhou, Yaling Wang, Wenxi Yu, Yonggang Wang, Lina Tang, Aina He, Zan Shen, Yang Yao, Haiyan Hu, Xin Liu, Jianjun Zhang

**Affiliations:** ^1^ Department of Oncology, Shanghai Jiao Tong University Affiliated Sixth People’s Hospital, Shanghai, China; ^2^ School of Artificial Intelligence, Guangxi University for Nationalities, Nanning, China; ^3^ Department of Pharmacy, Shanghai Jiao Tong University Affiliated Sixth People’s Hospital, Shanghai, China; ^4^ Department of Oncology, The Eighth People’s Hospital of Shanghai, Shanghai, China; ^5^ Department of Medical Oncology, Fudan University Shanghai Cancer Center, Shanghai, China; ^6^ Department of Oncology, Shanghai Medical College, Fudan University, Shanghai, China

**Keywords:** ailanthone, osteosarcoma, cancer metabolism, serine biosynthesis, PHGDH, multiomics analysis

## Abstract

Osteosarcoma (OS) is the most common primary bone sarcoma, chemoresistance becomes an obstacle to its treatment. Metabolic reprogramming is a hallmark of malignancy, targeting the metabolic pathways might provide a reasonable therapeutic strategy for OS. Here we demonstrated that Ailanthone (AIL), a major component of the Chinese medicine Ailanthus altissima, significantly suppressed OS cell growth *in vitro* and *in vivo*. Furthermore, AIL dose-dependently inhibited cell migration and invasion, induced cell cycle arrest and apoptosis in OS cells. Combined transcriptomics, proteomics and metabolomics analyses revealed that AIL induced widespread changes in metabolic programs in OS cells, while the serine biosynthetic pathway (SSP) was the most significantly altered pathway. qRT-PCR and Western blot assay confirmed that the transcript and protein levels of the SSP genes (*PHGDH*, *PSAT1* and *PSPH*) were downregulated dose-dependently by AIL. In addition, we found out that many downstream pathways of the SSP including the one-carbon pool by folate, purine metabolism, pyrimidine metabolism, DNA replication and sphingolipid metabolism were downregulated after AIL treatment. In the revere test, *PHGDH* overexpression but not exogenous serine supplementation clearly attenuated the effects of AIL on OS cells. Taken together, AIL exerts antitumor effects on OS through mediating metabolic reprogramming, at least in part, by suppressing the SSP. Our findings suggest that AIL could emerge as a potential therapeutic strategy in OS.

## Introduction

Osteosarcoma (OS) is the most common primary bone sarcoma, affecting mainly adolescents and young adults (1). The survival of patients with OS has improved considerably with the advent of multi-drug chemotherapy regimen including high-dose methotrexate, doxorubicin, cisplatin and ifosfamide since the late 1970s (2). However, frequent acquisition of drug-resistance becomes an obstacle to its treatment, patients who do not respond to chemotherapy have an overall survival of only 20-25% at 5 years (3, 4). During the past 30 years, OS therapy and prognosis had only modest improvements (5). New alternative anticancer agents for OS are in urgent need.

Metabolic reprogramming is a hallmark of malignancy (6). This metabolic alteration allows tumors to achieve excessive cell growth, proliferation, metastasis potential, as well as drug-resistance (7). Ongoing studies have revealed that the origin and development of OS are associated with alterations in metabolic pathways. For instance, OS cells have higher mitochondrial biogenesis compared to normal osteoblasts (8). Metabolic shift from oxidative phosphorylation to glycolysis promotes proliferation in osteosarcoma (9) while a higher glycolysis rate of OS cells confers to their cisplatin-resistant property (10). Recent studies reported that activation of the serine biosynthetic pathway (SSP) drives oncogenesis and contributes to tumor growth (11–13). The upregulation of the first rate-limiting enzyme of the SSP, 3-phosphoglycerate dehydrogenase (PHGDH), has been identified in a large subset of cancers (13–20). OS cell lines including Saos-2, U2-OS, MG63, MNNG and NOS1 were found to have significantly higher PHGDH protein expression than that of mesenchymal cells. Clinically, higher PHGDH protein expression was correlated with poorer outcomes (21). Given the important role of metabolic reprogramming in OS, targeting the metabolic pathways might provide a new therapeutic strategy for this disease.

In this study, we identified that Ailanthone (AIL), a quassinoid extracted from the traditional Chinese medicine plant Ailanthus altissima (22), exhibited significant antitumor activity in OS *in vitro* and *in vivo*. Mechanistically, combined transcriptomics, proteomics and metabolomics analysis revealed that AIL induced widespread changes in metabolic programs in OS cells. In particular, the SSP was the most significantly affected pathway by AIL treatment. Moreover, many downstream pathways of the SSP were downregulated. In the reverse test, *PHGDH* overexpression clearly reduced the inhibitory effects of AIL on OS cells, revealing a mechanistic link between AIL and the SSP. Overall, our findings indicate that AIL suppresses OS cell growth through mediating metabolic reprogramming, suggesting that AIL could be used as a promising therapeutic candidate in OS treatment.

## Materials and Methods

### Cell Culture and Proliferation Assay

Human Saos-2 and U-2OS OS cells were purchased from the Cell Bank of Type Culture Collection of the Chinese Academy of Sciences (Shanghai, China). The cell lines were routinely cultured in DMEM (#8119283, Gibco, Carlsbad, CA, USA) supplemented with 10% fetal bovine serum (FBS, #10270-106, Gibco), 100 μg/mL streptomycin, and 100 U/mL penicillin at 37°C in an incubator with a constant airflow of 5% CO_2_ and 95% O_2_. As for reverse test, the above-mentioned medium was supplemented with extra 400 μM serine (#GA10157, GlpBio Technology, CA, USA) in the serine supplementation group. AIL (#GC31742, molecular weight, 376.4, purity > 98.00%, GlpBio Technology) was dissolved to 10mM in dimethyl sulfoxide (DMSO, #D4540, Sigma-Aldrich, St. Louis, MO, USA) and stored at -80°C. The final concentrations were 0.039-10 μM. Cell proliferation assay was performed using Cell Counting Kit-8 (CCK-8, #96992, Dojindo Molecular Technologies, Dojindo, Japan). The absorbance value was measured at a wavelength of 450 nm, with the reference wavelength set at 630 nm. Data were collected from three independent experiments, and the AIL-induced cell growth inhibition rate was calculated by comparison to untreated control cells.

### Wound Healing Assay

The migration ability of OS cells was detected using a wound healing assay as outlined previously (23). Briefly, OS cells in a 6-well plate were carefully scratched using 10 μL sterile pipette tips. Subsequently, the cells were washed twice and were cultured in DMEM with different concentrations (0, 0.01, 0.05 and 0.1 μM) of AIL for 24 h. Images were then taken to determine the width of the wound using a microscope (10× objective) (1×71, Olympus).

### Transwell Assay

Transwell assays were performed using Boyden chambers (Corning Inc., Corning, NY, USA) as previously described (24). Briefly, OS cells were treated with AIL at various concentrations for 24h. Next, 1×10^5^ cells viable cells (confirmed by trypan blue exclusion) from each well were seeded in serum-free medium onto the Matrigel-coated upper chamber, while complete medium was added in the lower compartment as a chemoattractant. After incubation of 24 h, the cells that migrated through the filter were fixed and stained (23). The invading cells were evaluated in five random fields under the microscope, and images were obtained.

### Apoptosis Analysis

OS cells were seeded in 6-cm plates and incubated with different concentrations of AIL. After 24 h, all the cells including the floating and adherent ones were harvested and stained with Annexin V-fluorescein isothiocyanate (FITC) and propidium iodide (PI) apoptosis detection kit (#556547, BD Biosciences Pharmingen, San Diego, CA, USA) according to the manufacturer’s protocols. The resulting fluorescence was assessed by fluorescence-activated cell sorting scan (FACS) flow cytometry (Becton Dickinson, Mountain View, CA, USA) as reported previously (25).

### Cell Cycle Analysis

After being treated with different concentrations of AIL for 24 h, the OS cells were harvested and fixed with 70% ethanol overnight at -20°C and stained with PI. The DNA content was detected using a FACS flow cytometry (Becton Dickinson) according to the manufacturer’s protocols. The cell cycle distribution was determined using FlowJo software 8.7.1 (Tree Star, Inc., Ashland, OR, USA).

### TMT-Based Quantitative Proteomics

Tandem mass tag (TMT)-based quantitative proteomic analysis was carried out on Saos-2 cells treated with IC50 (0.20 μM) of AIL or DMSO for 24 h (3 biological replicates each group). A schematic description of the experimental design and data process strategy is presented in **Figure 5A**. SDT (4%SDS, 100mM Tris-HCl, 1mM DTT, pH7.6) buffer was used for cell lysis and protein extraction. Quantitative proteomic analysis was performed at Shanghai Applied Protein Technology Co., Ltd as described previously (26). Briefly, 100 μg peptide of each sample was labeled using TMT-6-plex Isobaric Label Reagent (Thermo Fisher Scientific, Waltham, MA, USA) according to the manufacturer’s instructions. Then, the labeled peptides were separated into 10 fractions using the High pH Reversed-Phase Peptide Fractionation Kit (Thermo Fisher Scientific). Each fraction was separated with EASY-nLC 1000 Liquid Chromatograph (Thermo Fisher Scientific) and then subjected to LC-MS/MS analysis on a Q Exactive HF-X mass spectrometer (Thermo Fisher Scientific) for 60 min. The MS raw data for each sample were searched using the MASCOT engine (version 2.2, Matrix Science, London, UK) embedded into Proteome Discoverer 1.4 software (Thermo Fisher Scientific) against the SwissProt human proteome database (20395 items, released on 2021.01.06) concatenated with reverse decoy database for identification and quantitation analysis. All identified proteins were determined using a false discovery rate (FDR) threshold of ≤ 0.01. The methods for protein quantification were as followed: first, the ratio of label signals was output and calculated as a ratio to the reference sample; secondly, the necessary normalization on protein median was performed to eliminate experimental error utilizing Correcting Experimental Bias function with built-in settings in Proteome Discoverer 1.4 software (Thermo Fisher Scientific); finally, the protein is quantified based on the median of the relative intensity of the unique peptide of each protein.

### RNA-Seq Analysis

Cell culture and AIL treatment were the same as those in quantitative proteomic analysis. RNA samples were prepared using TRIzol (#15596026, Invitrogen) and subjected to RNA-seq analysis by Genome Center, WuXi App Tec as reported previously (27). Generally, 1 μg of qualified RNA (RIN > 8.0) was used as input for library construction following the Illumina TruSeq RNA Sample Preparation protocol. Then, RNA libraries were sequenced on an Illumina NovaSeq 6000 platform, PE 2×150bp. The average data yield for each sample was 20M PE reads with % of Q30 bases > 90. Subsequently, RNA-seq reads of each sample were aligned to the human genome (build 38) using short reads aligner STAR (version 2.5.1b). Gene annotation and quantification were then performed using RSEM24 with GENCODE annotation (release 24: http://www.gencodegenes.org).

### Untargeted Metabolomic Relative Quantitative Analysis

Untargeted metabolomics profiling of Saos-2 cells treated with AIL or DMSO was performed using ultra-high-performance liquid chromatography (1290 Infinity LC, Agilent Technologies, USA) coupled with a quadrupole time-of-flight system (AB Sciex TripleTOF 6600, AB SCIEX, USA) at Shanghai Applied Protein Technology Co., Ltd. Briefly, the cell samples were collected in 15-mL Vacutainer tubes and then centrifuged for 15 minutes. Aliquots of the samples were stored at −80°C for use. Then the samples were thawed at 4°C, and 100-μL aliquots were mixed with 400 μL of cold methanol/acetonitrile (1:1, v/v) to remove the protein. The mixture was centrifuged for 20 minutes. The supernatants were dried in a vacuum centrifuge. Detailed experimental procedures for mass spectrometry and data process methods are described previously (28). After normalization to the total peak intensity, the data were exported to the SIMCA-P software for multivariate data analysis to screen for differential metabolites.

### qRT-PCR Assay

The mRNA levels of *PHGDH*, phosphoserine aminotransferase 1 (*PSAT1*) and phosphoserine phosphatase (*PSPH*) were detected using a PrimeScript RNA RT-PCR Kit (#Q711-03, Vazyme Biotech co., ltd, Nanjing, China) according to the manufacturer’s protocol. The primer pairs are shown in **Table 1**. The relative mRNA levels were calculated using the 2^−ΔΔCt^ method.

**Table 1:** The qRT-PCR primers.

	Forward	Reverse
PHGDH	5′-ATCTGCGGAAAGTGCTCATCA-3′	5′-GTGGCAGAGCGAACAATAAGG-3′
PSAT1	5′-CGCAGAAGAAGCCAAGAAGTT-3′	5′-TGGCTTGGACAGGAAGTTTGA-3′
PSPH	5′-AAAATCTGTGGCGTTGAGGAC-3′	5′-TGTGGGGGTTGCTCTGCTAT-3′
GAPDH	5′-AACGGATTTGGTCGTATTGGG-3′	5′-CCTGGAAGATGGTGATGGGAT-3′

### Western Blot Assay

Expression of proteins in OS cells was quantified using a BCA Protein Assay Reagent Kit (#23228, Thermo Fisher Scientific) as described previously (23). The rabbit polyclonal cleaved-Caspase-3 (#9664), BCL-2 (#3498), BAX (#5023), cyclinD1 (#55506) and CDK4 (#12790) antibodies were purchased from Cell Signaling Technology (Boston, USA). The PHGDH (#14719-1-AP), PSAT1 (#10501-1-AP) and PSPH (#14513-1-AP) antibodies were purchased from Proteintech Group, Inc. (Chicago, IL, USA), and the band density was quantified using ImageJ software 1.8.0 (National Institutes of Health, Bethesda, MD, USA).

### Construction of the Plasmids and Transfection

The *PHGDH* overexpression pcDNA3.1 plasmid was constructed by Genechem Co., Ltd. (Shanghai, China). The plasmid was transfected into Saos-2 and U-2OS cells using Lipofectamine 3000 (#L3000015, Invitrogen) following the manufacturer’s protocol.

### Mice Xenograft Experiment

The animal study was performed in accordance with the NIH Guide for the Care and Use of Laboratory Animals approved by the Scientific Investigation Board of Shanghai Jiao Tong University Affiliated Sixth People’s Hospital. 5×10^6^ Saos-2 cells were suspended in 75 μL serum-free DMEM and combined with 75 μL of Matrigel (#356235, Corning, NY, USA), and then the mixture was injected into the right hind thigh of 6-8 weeks NOD*-Prkdc^scid^ Il2rg^em1^/Smoc* (NSG) mice (Model organisms center, Shanghai, China). Cell line-derived xenografts were allowed to grow to an average volume of 100 mm^3^, then mice were intraperitoneally injected with AIL (2mg kg^-1^) or DMSO (as controls) every 3 days. Length and width of resulting tumors as well as weight of mice were measured every 5 days, and tumor volumes were calculated using the formula (length×width^2^)/2. Mice were sacrificed and tumors were harvested 30 days after treatment initiation.

### Bioinformatics Analysis and Statistics

For proteomics analysis, Perseus software (v1.6.5.0) was used. Differentially ex-pressed proteins (DEPs) were filtered at *P* < 0.05 and with a fold change of ≥ 1.2 or ≤ 0.83. Gene Ontology (GO) annotation for DEPs was performed using the DAVID Bioinformatics Resources 6.8 (https://david.ncifcrf.gov/). Moreover, proteome and transcriptome data were subjected to Gene Set Enrichment Analysis (GSEA) separately based on the whole gene set with the rank list of all the available expression values. C2.cp.kegg from the Molecular Signatures Database (MSigDB, v7.4) were selected as gene sets for the KEGG pathway enrichment analysis. Significantly enriched pathways were selected at a level of 0.05 norminal *P*-value and 0.25 FDR *q*-value. Subsequently, enriched pathways from proteome and transcriptome data were combined to determine the pathway that was most significantly affected by AIL treatment.

For metabolomics data, differentially expressed metabolites were determined using an unpaired Student’s t-test. The variable importance in the projection (VIP) value >1 and *P* < 0.05 was considered as statistically significant. The above-obtained metabolites were blasted against the online KEGG database to retrieve their Compounds and were subsequently subjected to enrichment analysis. Only pathways with *P*-values under a threshold of 0.05 were considered as significantly changed pathways.

The non-omics data analysis was performed using GraphPad Prism 7 (GraphPad Software Inc., CA, USA). *P* values were two-tailed, and *P* < 0.05 was considered statistically significant. Continuous variables are presented as the mean ± SD.

## Results

### AIL Inhibited Proliferation, Migration and Invasion of OS Cells *In Vitro*

**Figure 1:**
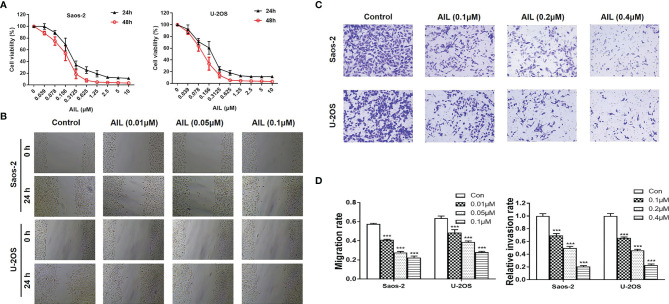
AIL inhibited proliferation, migration and invasion of OS cells. **(A)** After exposure to various concentrations (0.039-10 µM) of AIL for 24 h and 48h, the viability of Saos-2 and U-2OS cells was assessed with a CCK-8 assay. The cell viability decreased dose- and time-dependently. **(B)** After incubation with various concentrations of AIL, the wound healing assay indicated that AIL impaired wound healing dose-dependently. **(C)** Transwell assays showed that the invasion rates of OS cells decreased dose-dependently in the AIL groups. **(D)** Comparisons of the migration rates and relative invasion rates between the AIL and control groups are shown (^***^ *P* < 0.001 compared with the control).

The proliferation assay revealed that AIL significantly inhibited the proliferation of Saos-2 and U-2OS cells in a dose and time-dependent manner (**Figure 1A**). The IC50 at 24 h was determined to be 0.20 and 0.13 μM for the Saos-2 and U-2OS cells, respectively. Wound healing assays and Transwell assays were performed to determine the migratory and invasive abilities of OS cells. As shown in **Figures 1B, D**, compared with the migration rates of the control group, the migration rates of the Saos-2 and U-2OS cells treated with AIL were decreased dose-dependently. In the Transwell assay, AIL dose-dependently suppressed OS cell invasion (**Figures 1C, D**).

### AIL Suppressed Subcutaneously Xenografted OS Tumor Growth

**Figure 2:**
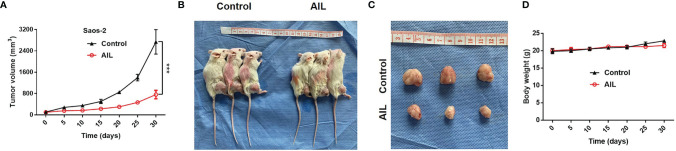
AIL inhibited subcutaneously xenografted tumor growth. **(A)** Saos-2 tumor-bearing mice were treated with AIL or DMSO intraperitoneally. The tumor volumes were measured every 5 days, growth curves for tumor proliferation were drawn accordingly (^***^ *P* < 0.001). **(B, C)** Mice were sacrificed and tumors were harvested (30 days after treatment), the mean tumor volume in AIL-treated mice and DMSO-treated mice was 2739 ± 796 mm^3^ and 760 ± 282 mm^3^, respectively (^***^ *P* < 0.001). **(D)** There was no significant difference in mice weight between the two groups.

To evaluated the efficacy of AIL *in vivo*, we treated Saos-2 xenografts in nude mice with AIL every 3 days. As a result, AIL significantly inhibited the increase of tumor volume in Saos-2 xenografts (**Figure 2A**). Mice were sacrificed after treatment, the mean tumor volume in AIL-treated mice was significantly lower than that in DMSO-treated mice (**Figures 2B, C**). In addition, there was no significant difference in mice weight between the two groups (**Figure 2D**), indicating that the dose of AIL was well tolerated.

### AIL Induced Apoptosis and Cell Cycle Arrest in OS Cells

**Figure 3:**
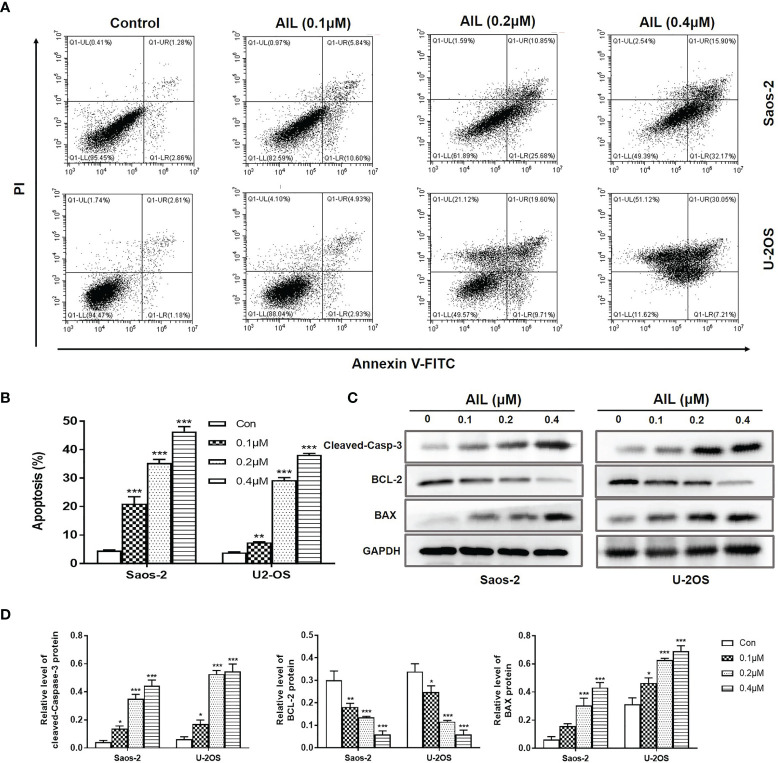
AIL induced apoptosis in OS cells. **(A)** Saos-2 and U-2OS cells were, respectively, treated with AIL at different concentrations (0, 0.1, 0.2 or 0.4 μ M) for 24 h. Then cells were stained with Annexin V-FITC and PI and then subjected to flow cytometry analysis of cell apoptosis. **(B)** Data of the percentage of apoptosis at various concentrations were pooled from **(A)**. **(C)** Apoptosis protein analysis. The protein levels of cleaved-Caspase-3, BCL-2 and BAX was determined by Western blot assay. **(D)** The protein levels were evaluated by ratio values quantified from protein bands of each marker versus GAPDH (^*^ *P* < 0.05, ^**^ *P* < 0.01, ^***^ *P* < 0.001 compared with the control).

**Figure 4:**
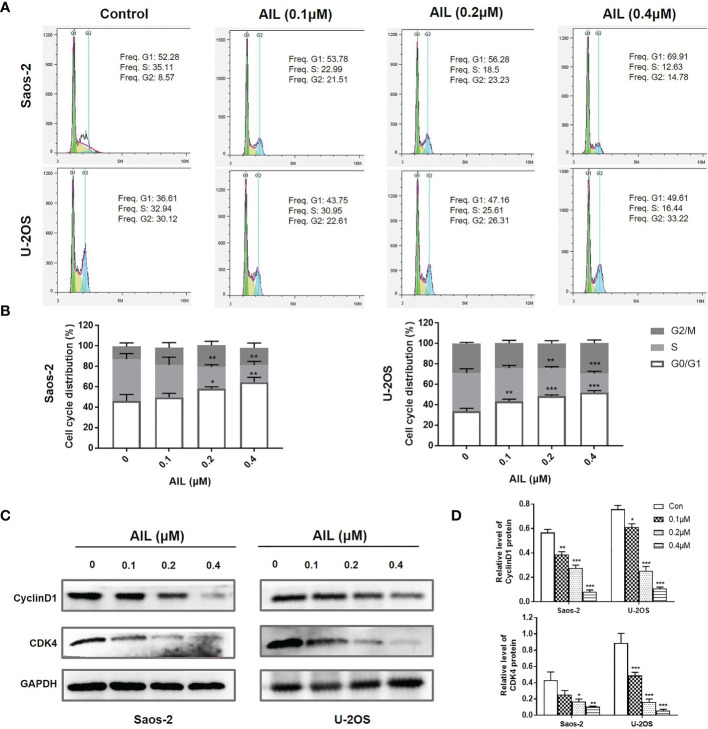
AIL blocked G1-S cell cycle transition in OS cells. **(A)** Cell cycle profiles were measured by flow cytometry following treatment of the cells with various concentrations of AIL (0, 0.1, 0.2 or 0.4 μ M) for 24 h. **(B)** The S phase was significantly decreased, while the G1 phase was significantly increased in Saos-2 and U-2OS cells after AIL treatment (^*^ *P* < 0.05, ^**^ *P* < 0.01, ^***^ *P* < 0.001 compared with the control). **(C)** The expression of cell cycle-related protein CyclinD1 and CDK4 was determined by Western blot assay. **(D)** The protein levels were evaluated by ratio values quantified from protein bands of each marker versus GAPDH (^*^ *P* < 0.05, ^**^ *P* < 0.01, ^***^ *P* < 0.001 compared with the control).

Since AIL showed a significant inhibitory effect on OS cell growth both *in vitro* and *in vivo*, we next explored the mechanism of action. As a result, AIL induced apoptosis in Saos-2 and U-2OS cells dose-dependently, which was supported by the downregulation of BCL-2 protein and upregulation of cleaved-Caspase-3 and BAX protein by Western blot assay (**Figure 3**). Cell cycle analysis showed that the S phase was significantly decreased, while the G1 phase was significantly increased in Saos-2 and U-2OS cells after AIL treatment (**Figures 4A, B**). G1-S transition related protein cyclinD1 and CDK4 were downregulated dose-dependently (**Figures 4C, D**). The results indicated that AIL blocked G1-S transition in OS cells in a dose-dependent manner.

### Treatment With AIL Led to Widespread Changes in Metabolic Programs

**Figure 5:**
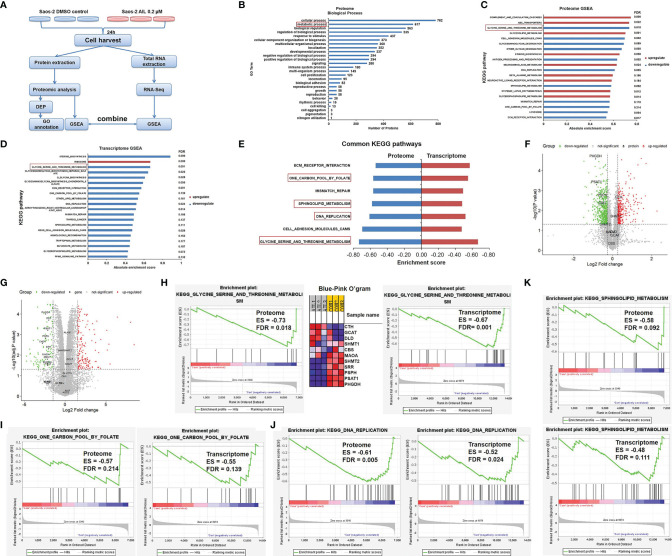
Proteomics and transcriptomics analysis of AIL treated Saos-2 cells identified the SSP as a key target. **(A)** Schematic overview of proteomics and transcriptomics analysis of Saos-2 cells treated with AIL or DMSO. **(B)** Gene ontology (GO) annotation showed that 617 (68.6%) out of the 899 differentially expressed proteins are involved in metabolic process. **(C)** GSEA of proteome data, showing the top 20 significant altered KEGG pathways after AIL treatment. **(D)** GSEA of transcriptome data, showing the top 20 significant altered KEGG pathways after AIL treatment. **(E)** Details of the 7 common KEGG pathways from proteome and transcriptome data. **(F)** Volcano plot of the global profile of proteins after AIL treatment, marked black proteins are involved in the serine biosynthetic or metabolic pathway. **(G)** Volcano plot of the global profile of mRNA after AIL treatment, marked black genes are involved in the serine biosynthetic or metabolic pathway. **(H)** Enrichment plot and Blue-Pink O’gram of the glycine, serine and threonine metabolism pathway from proteome and transcriptome data. **(I)** Enrichment plots of the KEGG one-carbon pool by folate pathway from proteome and transcriptome data. **(J)** Enrichment plots of the KEGG DNA replication pathway from proteome and transcriptome data. **(K)** Enrichment plots of the KEGG sphingolipid metabolism pathway from proteome and transcriptome data.

To investigate the possible network related to the inhibitory effect of AIL on OS cells, we performed a comparative proteomic analysis in Saos-2 cells treated with AIL or DMSO. In total, 6860 unique proteins (5684 proteins identified with at least two peptide fragments) were identified with high confidence (FDR ≤ 0.01). Among them, 6831 proteins were quantifiable (5674 proteins were quantifiable with at least two peptide fragments). 899 DEPs (342 upregulated and 557 downregulated, **Supplementary Table 1**) were filtered at *P* < 0.05 and fold change ≥ 1.2 or ≤ 0.83. The classification of DEPs by GO database annotation showed that 617 (68.6%) proteins are involved in the metabolic process (**Figure 5B**), suggesting that AIL treatment leads to widespread changes in metabolic programs in OS cells. To identify the specific signaling pathways in detail, GSEA was performed. As a result, 28 KEGG pathways were significantly enriched (**Figure 5C**). Furthermore, we performed RNA-Seq analysis to interrogate changes at transcript level in OS cells exposed to AIL. GSEA analysis revealed that 30 KEGG pathways were significantly enriched (**Figure 5D**). Then we combined the significantly enriched pathways from proteome and transcriptome data, we found out that 7 KEGG pathways were common (**Figure 5E**). Among the 7 common pathways, the serine biosynthetic or metabolic pathway was the pathway most significantly changed (**Figures 5C–G**). Notably, the GSEA Blue-Pink O’gram (**Figure 5H**) revealed that PHGDH, PSPH and PSAT1, the three rate-limiting enzymes in SSP, are the top three downregulated proteins. These bioinformatic results suggested that the SSP is a key target of AIL in OS cells. Serine is a precursor to folate, which is the principal donor of one-carbon units (29, 30), it serves as a major source of metabolic intermediates that are required for nucleotide synthesis. Serine is also the precursor to sphingolipids (29, 31–33). Interestingly, GSEA results indicated that the one-carbon pool by folate, DNA replication and sphingolipid metabolism pathways were among the 7 common pathways downregulated by AIL treatment (**Figures 5E, I–K**). These data further support the connection between the effects of AIL and the SSP.

### Treatment With AIL Led to Reduced SSP Expression

**Figure 6:**
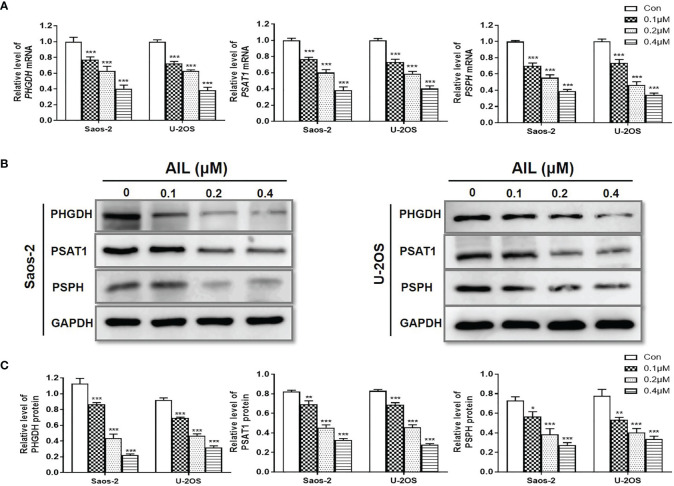
Treatment with AIL led to reduced SSP expression. **(A)** qRT-PCR revealed that the *PHGDH*, *PSAT1* and *PSPH* mRNA levels were downregulated by AIL dose-dependently in Saos-2 and U-2OS cells (^*^ *P* < 0.05, ^**^ *P* < 0.01, ^***^ *P* < 0.001 compared with the control). **(B)** The expression of PHGDH, PSAT1 and PSPH protein was downregulated by AIL dose-dependently in Saos-2 and U-2OS cells. **(C)** Data of relative protein level were pooled from three independent experiments (^*^ *P* < 0.05, ^**^ *P* < 0.01, ^***^ *P* < 0.001 compared with the control).

To validate the changes of SSP expression induced by AIL, we measured the expression of mRNA and protein of the SSP in Saos-2 and U-2OS cells using qRT-PCR and Western blot assay, respectively. In line with the RNA-Seq-based gene expression, all of the *PHGDH*, *PSAT1* and *PSPH* mRNA was downregulated significantly in both of the two cell lines after AIL treatment (**Figure 6A**). Similarly, protein expression of each enzyme in the SSP was confirmed to be downregulated by AIL dose-dependently (**Figures 6B, C**).

### Treatment With AIL Led to Reduced Intermediate Metabolites of the SSP and Its Downstream Pathways

**Figure 7:**
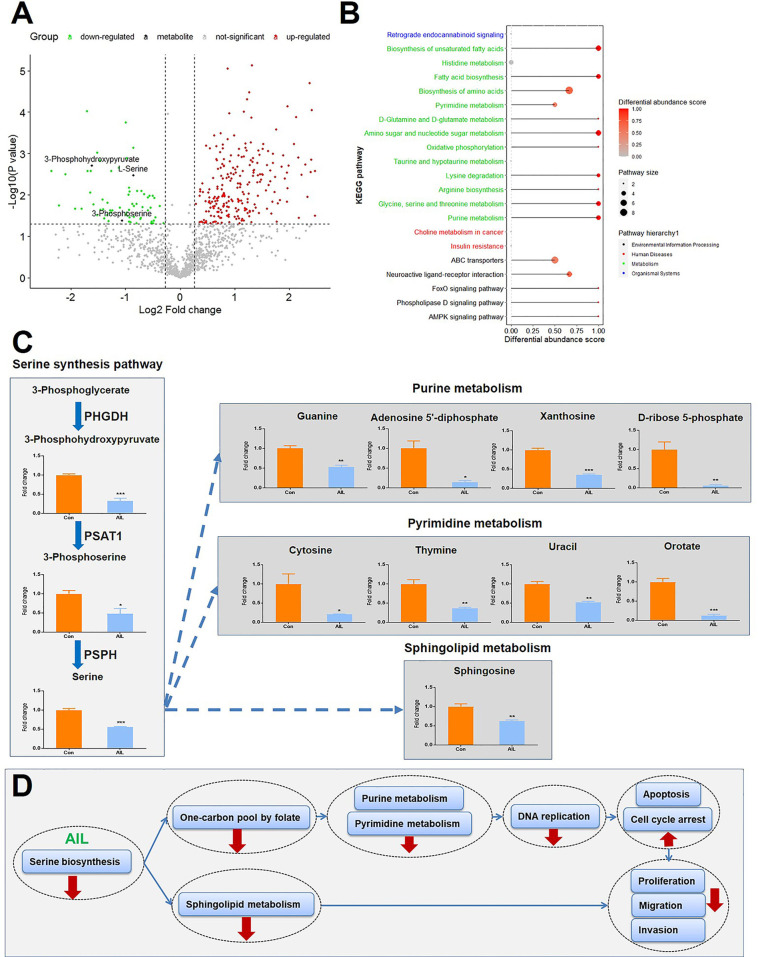
Treatment with AIL led to reduced intermediate metabolites of the SSP and its downstream pathways. **(A)** Volcano plot of the global profile of metabolites after AIL treatment, marked black metabolites are involved in the serine biosynthetic or metabolic pathway. **(B)** KEGG enrichment of differentially expressed metabolites, showing 21 significantly enriched pathways after AIL treatment. **(C)** Fold-changes in the intermediate metabolites of the SSP and its downstream pathways (^*^ *P* < 0.05, ^**^ *P* < 0.01, ^***^ *P* < 0.001 compared with the control). **(D)** Proposed schematic overview of the antitumor mechanism of AIL in OS.

Since the above-mentioned data suggested that the effects of AIL are associated with changes in metabolic programs, untargeted metabolomics with high resolution, high throughput, and highly sensitive technology was performed to execute a large‐scale detection of the metabolite features after AIL treatment (**Figure 7A**). As shown in **Figure 7B**, 21 KEGG pathways were significantly enriched, out of which, 13 were involved in metabolism and **Supplementary Table 2**. To be noted, glycine serine and threonine metabolism was one of the pathways that are changed the most. And then, we asked about the change of intermediate metabolites of the SSP. As expected, the relative levels of 3-phosphohydroxypyruvate, 3-phosphoserine and serine in OS cells were reduced significantly after AIL treatment (**Figures 7A, C**). Serine is important in metabolism in that it participates in the biosynthesis of purines, pyrimidines and sphingolipids. Interestingly, the intermediate metabolites of the downstream pathways of the SSP including the purine metabolism, pyrimidine metabolism and sphingolipid metabolism were also downregulated significantly (**Figure 7C**). These results are in line with the data at mRNA and protein levels. Taken together, we proposed that AIL inhibited proliferation, migration and invasion of OS cells by downregulating the SSP, further downregulating the pathways of one-carbon pool by folate, purine metabolism, pyrimidine metabolism, DNA replication and sphingolipid metabolism (**Figure 7D**).

### Overexpression of *PHGDH* but Not Exogenous Serine Supplementation Reversed the Effects of AIL on OS Cells

**Figure 8:**
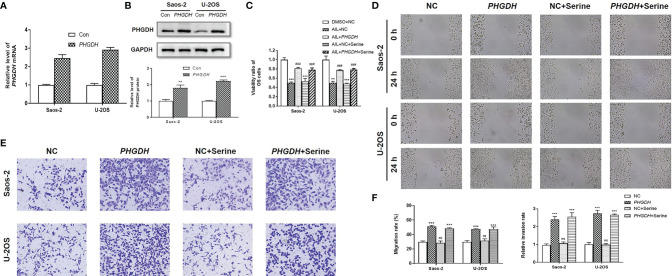
Overexpression of *PHGDH* but not exogenous serine supplementation reversed the effects of AIL on OS cells. **(A, B)** Overexpression efficiency of *PHGDH* was verified by qRT-PCR and Western blot assay. **(C)** OS cells were assigned to five groups: negative control (NC) treated with DMSO (DMSO + NC), NC treated with AIL (AIL + NC), *PHGDH* overexpression treated with AIL (AIL + *PHGDH*), NC treated with AIL and serine (AIL + NC *+* Serine), *PHGDH* overexpression treated with AIL and serine (AIL + *PHGDH +* Serine). The viability ratio of Saos-2 and U-2OS cells in the AIL + *PHGDH* group and AIL + *PHGDH +* Serine group but not in the AIL + NC *+* Serine group was significantly higher than that in the AIL + NC group (^**^ *P* < 0.01, ^***^ *P* < 0.001 compared with the DMSO + NC group, ^###^ *P* < 0.001 compared with the AIL + NC group). **(D)** The wound healing assay revealed that overexpression of *PHGDH* but not exogenous serine supplementation reversed the suppressive effect of AIL on the migration of OS cells. **(E)** The Transwell assay demonstrated that overexpression of *PHGDH* but not exogenous serine supplementation reversed the suppressive effect of AIL on OS cell invasion. **(F)** Comparisons of the migration rates and relative invasion rates between the NC group and the other groups are shown (^***^ *P* < 0.001, ^ns^ *P* > 0.05).

To further determine the functional importance of the SSP in AIL effects, the *PHGDH* in OS cells were overexpressed by the treatment with IC50 of AIL (**Figures 8A, B**). Besides, we supplemented exogenous serine in the media to explore if downregulation of serine biosynthesis by AIL can be compensated for with exogenous serine. As expected, the viability of Saos-2 and U-2OS cells significantly increased in the *PHGDH* overexpression group compared with that in the negative control group. However, the inhibitory effect of AIL on OS cell proliferation can’t be reversed by exogenous serine supplementation. Moreover, exogenous serine supplementation didn’t affect the reverse effect of *PHGDH* overexpression (**Figure 8C**). Similarly, the wound healing assay and Transwell assay revealed that overexpression of *PHGDH* but not exogenous serine supplementation attenuated the suppression of AIL on the migration and invasion of OS cells (**Figures 8D–F**). These data demonstrated that AIL suppresses OS cells through downregulating the serine *de novo* biosynthesis but not exogenous serine importation.

## Discussion

In the past decade, an increasing number of studies have focused on AIL due to its antitumor activity in a variety of malignances, including leukemia, lung, breast, gastric, liver and prostate cancer (22, 34–38). However, the effects of AIL on OS remain largely unexplored. In this study, we found that AIL dose-dependently inhibited cell proliferation, migration and invasion while inducing G1-S cell cycle arrest and apoptosis in Saos-2 and U-2OS cells. Our results were similar to that of researches in gastric and liver cancer (37, 38).

The antitumor mechanism of AIL differs a lot in different cancer types (39). To explore the underlying networks that are regulated by AIL in OS cells, we performed combined transcriptomics, proteomics and metabolomics analysis. The results revealed that AIL induced widespread changes in metabolic programs in OS cells. In particular, AIL treatment led to significantly reduced SSP at transcript, protein, as well as metabolite levels. Then we selected the SSP for further validation, not only because the SSP was the pathway most significantly affected by AIL, but also because the SPP was confirmed to be abnormally activated in OS and serine is a central hub of cancer metabolism (21). The SSP represents a crucial turning point in glucose conversion (40). It converts the glycolytic intermediate, 3-phosphoglycerate, to serine *via* three sequential enzymes PHGDH, PSAT1 and PSPH. Serine is then converted to glycine, concomitantly charging the folate pool with one-carbon units (29, 30, 41–43). Both glycine and folate one-carbon units are used to make nucleotides. Serine is also required for the synthesis of sphingolipids, a major component of cellular membranes (31–33). Moreover, serine biosynthesis affects cellular antioxidative capacity, thus maintaining tumor homeostasis (11, 20). Hyperactive SPP frequently enables cancer cells to survive and proliferate irrespective of the availability of exogenous serine (44, 45).

In the subsequent experiments, the transcript and protein levels of the SSP genes (*PHGDH*, *PSAT1* and *PSPH*) were confirmed to be downregulated dose-dependently by AIL in OS cells using qRT-PCR or Western blot assay. To validate the key role of the SSP, the reverse test was performed. As expected, after overexpressing *PHGDH*, the suppression of AIL on OS cell proliferation, migration, and invasion was clearly reduced. We reason that inhibition of *de novo* serine biosynthesis causes failure to fuel the subsequent DNA synthesis and cellular membrane production, which are essential to cancer proliferation. This proposed mechanism was further supported by the downregulation of several downstream pathways of the SSP and the cell cycle arrest in OS cells after AIL treatment. Similarly, Ni et al. reported that AIL inhibited non-small cell lung cancer cell growth through repressing DNA synthesis (35). Proliferating cells can import serine exogenously or through *de novo* serine biosynthesis *via* the SSP. However, we found that exogenous serine supplementation didn’t reverse the effects of AIL on OS cells. We then asked why *PHGDH* inhibition cannot be compensated for with exogenous serine, one possible explanation is the relatively low-baseline mRNA expression of the serine transporter *SLC1A5* in OS cells, as shown in data from the Cancer Cell Line Encyclopedia (46). Indeed, the detailed mechanistic understanding of why some cancer cells are addicted to serine synthesis despite the availability of extracellular serine for import remains unclear (47).

The importance of the metabolic pathways is underlined in OS by the fact that the inhibitor of dihydrofolate reductase methotrexate is one of the most effective chemotherapeutic agents for OS (48). Given the feasibility of regimens targeting the folate pathway in OS, inhibition of the upstream SSP deserves to be explored as a possible replacement for the toxic high-dose methotrexate, especially for patients who do not benefit from methotrexate.

In summary, our study demonstrated that AIL significantly inhibited OS cell proliferation, migration and invasion through targeting metabolic pathways, at least in part, by suppressing the SSP. AIL is worthy to be further explored as a candidate for the treatment of OS.

## Data Availability Statement

The datasets presented in this study can be found in online repositories. The names of the repository/repositories and accession number(s) can be found in the article/**Supplementary Material**.

## Ethics Statement

The animal study was reviewed and approved by the Scientific Investigation Board of Shanghai Jiao Tong University Affiliated Sixth People’s Hospital.

## Author Contributions

JZ, XL and HH designed the research. YZha, RG and XS performed the *in vitro* research. YZha and JL completed the animal study. YL and YZho contributed to bioinformatic analysis. YgW, LT and AH performed the non-omics data analysis. WY and YlW prepared the figures. ZS and YY polished the English translation. XL and JZ wrote the paper. All authors contributed to the article and approved the submitted version.

## Funding

This work was supported by grants from the National Natural Science Foundation of China (82072967), Shanghai Senior Integrative Chinese and Western Medicine Talents Program (ZY(2018-2020)-RCPY-2017) and Shanghai Pujiang Program (21PJD051).

## Conflict of Interest

The authors declare that the research was conducted in the absence of any commercial or financial relationships that could be construed as a potential conflict of interest.

## Publisher’s Note

All claims expressed in this article are solely those of the authors and do not necessarily represent those of their affiliated organizations, or those of the publisher, the editors and the reviewers. Any product that may be evaluated in this article, or claim that may be made by its manufacturer, is not guaranteed or endorsed by the publisher.
